# Genetic Diversity in the Italian Holstein Dairy Cattle Based on Pedigree and SNP Data Prior and After Genomic Selection

**DOI:** 10.3389/fvets.2021.773985

**Published:** 2022-01-13

**Authors:** Michela Ablondi, Alberto Sabbioni, Giorgia Stocco, Claudio Cipolat-Gotet, Christos Dadousis, Jan-Thijs van Kaam, Raffaella Finocchiaro, Andrea Summer

**Affiliations:** ^1^Dipartimento di Scienze Medico-Veterinarie, University of Parma, Parma, Italy; ^2^Associazione Nazionale Allevatori della Razza Frisona Bruna e Jersey Italiana, Cremona, Italy

**Keywords:** inbreeding, runs of homozygosity, effective population size, cattle, genomic selection, sustainability

## Abstract

Genetic diversity has become an urgent matter not only in small local breeds but also in more specialized ones. While the use of genomic data in livestock breeding programs increased genetic gain, there is increasing evidence that this benefit may be counterbalanced by the potential loss of genetic variability. Thus, in this study, we aimed to investigate the genetic diversity in the Italian Holstein dairy cattle using pedigree and genomic data from cows born between 2002 and 2020. We estimated variation in inbreeding, effective population size, and generation interval and compared those aspects prior to and after the introduction of genomic selection in the breed. The dataset contained 84,443 single-nucleotide polymorphisms (SNPs), and 74,485 cows were analyzed. Pedigree depth based on complete generation equivalent was equal to 10.67. A run of homozygosity (ROH) analysis was adopted to estimate SNP-based inbreeding (F_ROH_). The average pedigree inbreeding was 0.07, while the average F_ROH_ was more than double, being equal to 0.17. The pattern of the effective population size based on pedigree and SNP data was similar although different in scale, with a constant decrease within the last five generations. The overall inbreeding rate (ΔF) per year was equal to +0.27% and +0.44% for F_ped_ and F_ROH_ throughout the studied period, which corresponded to about +1.35% and +2.2% per generation, respectively. A significant increase in the ΔF was found since the introduction of genomic selection in the breed. This study in the Italian Holstein dairy cattle showed the importance of controlling the loss of genetic diversity to ensure the long-term sustainability of this breed, as well as to guarantee future market demands.

## Introduction

The Holstein dairy cattle have been bred in a pioneering effort to increase milk yield over the last century while, more recently, the emphasis on functional, longevity, and fertility traits has grown. This breed is counted in over 150 countries, and it is the most common dairy cattle breed worldwide (1). Despite its abundance, concerns on the decreasing level of genetic diversity of the Holstein populations have been recently raised in several countries (2–4). These current alarms stem from the intense directional selection practiced in the last century, which has caused loss of genetic variability. The nineteenth century harbored the adoption of selection theories, such as the selection index theory (5), and advanced statistical methods, such as the best linear unbiased prediction (BLUP) (6), promoting a remarkable improvement in the genetics of dairy cattle breeds. In addition, the implementation of artificial insemination (AI) over 80 years ago boosted the impact of elite sires worldwide, thus allowing a superior genetic gain per generation (7). In the last 20 years, the advances in high-throughput genotyping procedures allowed the development of single-nucleotide polymorphism (SNP) chips at reasonable price, thereby determining the application of genomic selection (GS) based on SNP arrays (8) in several Holstein breeding programs in different countries (4, 9–11). A recent study evaluated the impact of GS implementation on the US Holstein and showed an outstanding increase in the annual genetic gain rate, ranging from 50 to 100% for yield traits and from 200 to 300% for fitness traits (12). The increase in the reliability of genomic breeding values (GEBVs) over traditional estimated breeding values (EBVs) for young bulls is quite remarkable, reaching up to a 20% increase for some traits (11). Since young bulls can now be selected as sires based on their GEBV at a very early stage, generation intervals (GIs) have been shortened significantly (2, 4, 13, 14). However, there is increasing evidence that the maximization of genetic gain is counterbalanced by a reduction of genetic diversity within the breed. Moreover, it has been shown that the annual inbreeding rate has increased in several dairy cattle populations after the implementation of GS (2, 4, 13). This may be partly attributed to the fact that, while the overall number of sires of bulls has increased since the introduction of GS, the number of popular bulls (siring half of the young bulls entering AI) has remained fairly stable (15).

Managing genetic diversity determines the long-term sustainability of the livestock production sector. The impact of climate change on livestock, market demand fluctuations, and the increase in human population urgently require a sufficient reservoir of genetic diversity (16). It is therefore crucial to evaluate the overall pool of genetic diversity in both commercial and local breeds to preserve biodiversity. Traditionally, the study of genetic diversity relies on pedigree information, where inbreeding can be estimated as the probability of an individual to have two identical alleles by descent (17). Analyses at the pedigree level are particularly effective to evaluate the state of genetic diversity in small and underdeveloped populations with limited financial resources that cause the unavailability of more advanced technologies such as genomic data (18–21). However, those estimates highly depend on quality and depth of the pedigree information and rely on the assumption that no relationship exists among founder animals; hence, pedigree-based inbreeding estimates tend to underestimate actual inbreeding coefficients (3, 22). The advent of genomics allowed researchers to gain insights into genetic diversity by using genotype data (23). One of the most well-established methods to detect within-breed loss of genotypic diversity is the run-of-homozygosity (ROH) detection (24). The ROHs are long consecutive homozygous segments distributed across the genome, which arise from identical-by-descendent haplotype (25, 26). Hence, ROHs have been commonly used to estimate genomic inbreeding (F_ROH_) in several species such as cattle (13, 27–29), horses (30–33), pigs (34, 35), sheep (36, 37), and goats (38, 39). In contrast to pedigree-based inbreeding estimates, F_ROH_ can capture the variation due to Mendelian sampling and linkage during gamete formation (23).

In Italy, the most reared dairy cattle breed is the Italian Holstein, counting for more than 1,000,000 live animals and about 9,500 breeders, with an average of 10,386 kg of milk produced per lactation/cow in 2020 (40). However, few studies have evaluated the level of genetic diversity in this breed by studying SNP data. More precisely, previous reports on genomic inbreeding in the Italian Holstein were based on 50K SNP data in 2,093 bulls (41) and in a set of 96 animals (42). Moreover, a recent study that aimed to evaluate the presence of genomic divergence in Italian Holstein cows bred for different production chains calculated the inbreeding based on ROH in 1,000 Italian Holstein cows (43). Nevertheless, to the best of the authors' knowledge, the genetic variability in the Italian Holstein has not been fully explored, especially using a large database on the female side and with deep historical data. Thus, in this study, we aimed to investigate the genetic diversity using data from Italian Holstein cows born between 2002 and 2020. The specific aims of the study were to (i) calculate the inbreeding based on pedigree and genotype data, (ii) evaluate changes in the effective population size throughout generations, and (iii) test the effect of GS on genetic diversity and GI.

## Materials and Methods

Records used in this study were obtained from archived data provided by the Italian National Association of Holstein, Brown Swiss, and Jersey Breeders (ANAFIBJ), and as such, no approval was required for animal experimental purposes from the Animal Care Committee unit of the University of Parma. The consent for data use was obtained by ANAFIBJ.

### Pedigree Data

Pedigree records for the genotyped animals were provided by ANAFIBJ. Pedigree information consisted of 393,607 individuals born between 1898 and 2020 with 26,226 males and 367,381 females over 24 generations of pedigree depth. To evaluate the role of pedigree depth in the inbreeding estimates, the complete generation equivalent (CGE) was calculated using the optiSel package (44) in R software (45).

### Genotype Data

A total of 95,497 genotyped Italian Holstein cows born between 2002 and 2020 were available for this study. Cows were genotyped with a variety of SNP panels, ranging from low- to high-density panels. The animals genotyped with low-density panels were imputed to medium density (85K) using PedImpute (46). To guarantee high accuracy during the imputation pipeline, females were retained for this study only when both sire and sire of the dam were (i) genotyped and (ii) used in the imputation pipeline. Quality control (QC) excluding poorly genotyped and faulty data was performed on the 29 autosomal chromosomes by using PLINK v1.90 (47). The QC was based on the following criteria: call rate of <95%, parent–offspring SNP mismatch of <0.01, minor allele (<0.01) and genotype (<0.001) frequencies, and extreme deviation from the Hardy–Weinberg equilibrium (*P* < 0.005).

### Pedigree and SNP-Based Inbreeding Coefficients

The pedigree-based inbreeding coefficient (F_ped_) was defined as the probability of an individual presenting two identical alleles by descent (48). The coefficients were computed by using the optiSel (44) package in R (45). The SNP-based inbreeding coefficient was derived by means of ROH assessments, a segment-based approach. The ROH segments were detected by using the detectRUNS package (49) in R (45) and defined as follows: (i) at least 15 SNPs in a run, (ii) a minimum length of a run equal to 1 Mb, (iii) a maximum distance between consecutive SNPs in a window of 500 kb, (iv) a lower density limit of one SNP per 100 kb, and (v) a maximum of one missing and one heterozygous SNP being allowed in a run. The genomic inbreeding coefficient (F_ROH_) was calculated as follows (50):


(1) $$F_{ROH}\;=\;\frac{\sum L_{ROH}}{L_{AUTO}}\;$$

with L_ROH_ being the sum of the length of ROHs per cow and L_AUTO_ the total length of the autosomal genome covered by SNPs (in this study 2.48 Gbp). The correlation between F_ped_ and F_ROH_ was calculated by means of Pearson's product–moment correlation (r). To compare the variability of F_ped_ and F_ROH_, we calculated the coefficient of variation of these two measurements as the ratio between the standard deviation of inbreeding and its overall mean. Moreover, the r_Fped−FROH_ was estimated per year. To evaluate the effect of CGE on the relationship between F_ped_ and F_ROH_, the database was divided into cows with CGE ≤ 10 (*N* = 21,028) and cows with CGE > 10 (*N* = 53,457). Based on the hypothesis that ROH length reflects the chronological time points when inbreeding happened, the genomic inbreeding was expressed separately for six length ROH categories to differentiate old and recent inbreeding (1 > ROH ≤ 2, 2 > ROH ≤ 4, 4 > ROH ≤ 8, 8 > ROH ≤ 16, 16 > ROH ≤ 32, and ROH > 32 Mbp) by dividing the database into four birth-year classes, considering an average GI of 5 years as found from the pedigree data.

### Effective Population Size

The effective population size (Ne) of an actual population can be defined as the size of a hypothetical ideal population resulting in the same amount of genetic drift as is present in the real population (48). In this study, we estimated the historical and recent Ne based on both pedigree and SNP data. The optiSel (44) package in R (45) was used to estimate the Ne based on pedigree data from 1960 to 2020. The SNeP v.1.1 software was used to estimate the trends of historical Ne based on linkage disequilibrium (LD) on SNP data (51) by using animals born in the latest year (677 cows in 2020). The Ne was estimated for the latest 30 generations as the first introduction of the Dutch Friesian in Italy dates back to 1,870 followed by North American Holstein Friesians from 1923. Since different methods for Ne estimation based on LD are available in the literature, two analyses were performed by (a) using default settings, except for the recombination rate, which was inferred following the Sved and Feldman method (52), and the mutation rate in cattle [α = 2.2 (53)]; and (b) including a restriction on the maximum distance used to calculate LD. The latter parameterization, together with the Sved and Feldman mutation rate modifier, allowed us to estimate Ne for the most recent generations (53, 54).

### The Role of GS on Genetic Diversity and GI

The rate of inbreeding (ΔF) per year was calculated as the inverse of the slope of the regression of ln(1−x) on the year of birth, where *x* was equal to the average of the parameter each year (F_ped_ and F_ROH_) (2). The annual rate was multiplied by the GI (in the Italian Holstein being equal on average to 5 years as shown in this study) to obtain the rate per generation (ΔF_gen_). To assess the effect of different selection strategies on genetic diversity in the Italian Holstein breed, i.e., classical progeny testing (PTS) vs. GS, we divided the database into two 5 year birth cohorts by considering all animals born before (2006 to 2010) and after the introduction of GS (2015 to 2019). Then, we tested the equality of PTS and GS means. Moreover, to evaluate the impact of the introduction of GS on genetic diversity, we used the following linear model, using the R function lm (45):


(2) $$Y_{i}=\left\{\begin{array}{c} \alpha_{1}+\beta_{PTS}x_{i}\;+\;\varepsilon_{i\;},2006\;\leq \;x_{i}\;\leq \;2010\\ \alpha_{1}+(\beta_{PTS}+\delta )x_{i}\;+\;\varepsilon_{i\;},2015\;\leq \;x_{i}\;\leq \;2019 \end{array}\right\}\;$$

where *Y*_*i*_ is the variable of interest for each cow *i* (F_ped_ and F_ROH_), *x*_*i*_ is the birth year of each cow *i*, and β_*PTS*_ is the associated coefficient of regression if cow *i* was born in the PTS cohort or β_*PTS*_ + δ if cow *i* was born in the GS cohort. The impact of GS on the inbreeding rate was measured with the δ coefficient. Analysis of variance was used to test the significance of the δ value. The relative change (RC) of the slopes of regression before and after GS was computed as *RC* = δ/β_1_, with β_1_ being the slope in the second evaluated period. The assessment of the RC allowed the evaluation of slope value through time (13). The effect of the introduction of GS was also evaluated via the length of the GI. The GI, defined as the average age of parents when their offspring were born, was calculated for all the four selection pathways (sire of bulls, dam of bulls, sire of cows, and dam of cows) using the 393,607 individuals present in the pedigree. The GI was estimated using the Pedig software (55).

## Results

### Pedigree and SNP-Based Inbreeding Coefficients

**Figure 1:**
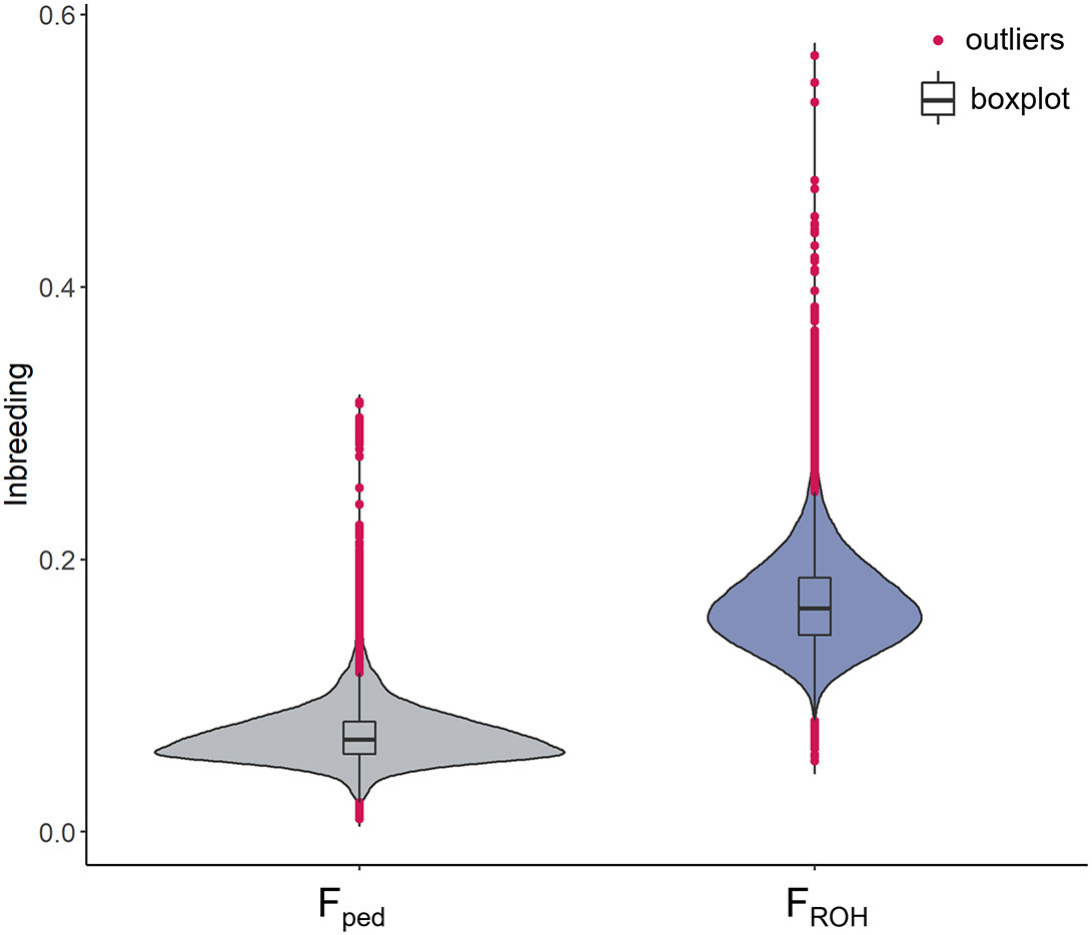
Violin plots of the inbreeding coefficients in the Italian Holstein cows. On the left side the inbreeding based on pedigree (F_ped_) and on the right the inbreeding based on ROH data (F_ROH_). Black horizontal line within the boxplot represents the median. Extreme values (above and below the mean ± 3 SD) are presented in magenta.

**Figure 2:**
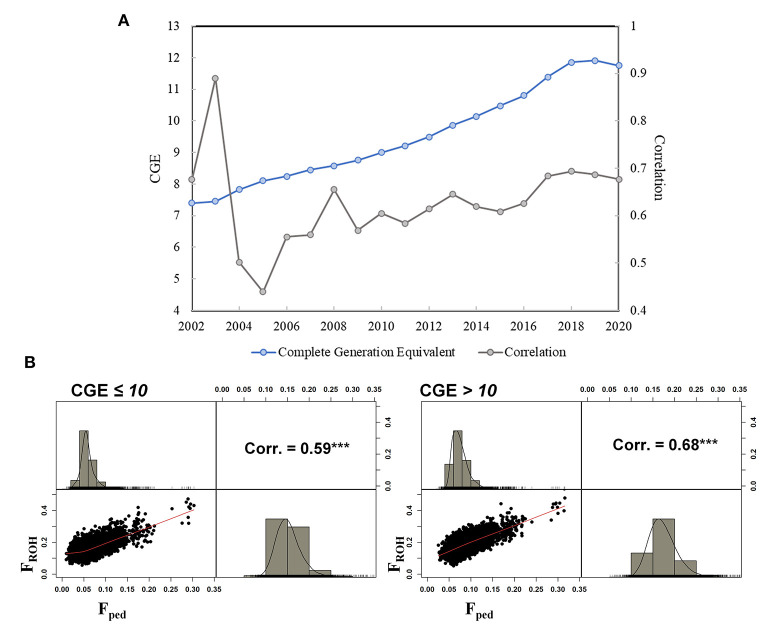
**(A)** Pearson correlation between F_ped_ and F_ROH_ is shown in gray color and the CGE from 2002 to 2020 in Italian Holstein cows is shown in light blue color, **(B)** Pearson correlation between F_ped_ and F_ROH_ dividing the sample in two subgroups based on CGE (CGE ≤ 10 on the left side, and CGE > 10 on the right side). The Pearson correlation is shown above diagonal, the scatterplot below the diagonal and the density plots of inbreeding coefficients measured by ROH (FROH) and pedigree data (Fped) in the Italian Holstein dairy cows are shown on the diagonal.

A total of 84,443 SNPs and 74,485 cows were kept after QC. The average genotyping call rate was 0.99, and the average pedigree depth based on CGE was equal to 10.67 (SD = 1.12). The cows descended from 3,058 sires and 59,377 dams. In total, over 50% of the cows was born between 2016 and 2020 (last 5 years), whereas 34,286 cows were born between 2002 and 2015. The mean F_ped_ was equal to 0.07 (SD = 0.02) ranging between 0.01 and 0.32 (CV = 0.29). The F_ROH_ mean value was more than doubled (0.17; SD = 0.03), with a minimum and maximum of 0.05 and 0.50, respectively (CV = 0.20). A total of 839 cows showed an F_ped_ exceeding the mean + 3 SD (corresponding to F_ped_ ≥ 0.13), which were defined as highly inbred females. In the case of the F_ROH_, 507 cows presented an inbreeding higher than the mean + 3 SD (F_ROH_ ≥ 0.27) (Figure 1). Approximately 23% of the highly inbred cows were in common from the comparison between the two methodologies. The Pearson correlation between F_ped_ and F_ROH_ was equal to 0.68 (confidence interval: 0.676–0.683), *P*-value < 2.2e−16) as shown in Supplementary Figure 1. The average CGE in the entire database was equal to 10.06, showing an increase throughout the studied period from 2002 (average CGE = 7.5) to 2019 (average CGE = 11.9) (Figure 2A). The correlation ranged from 0.44 in 2005 (*N* = 136) to 0.89 in 2003 (*N* = 16); however, those estimates might be an artifact produced by the limited number of cows for those years; thus, they should be considered with caution. Since 2010, the number of born and genotyped cows per year has been above 1,000, and the r_Fped−FROH_ was steadier, ranging between 0.58 and 0.69. The r_Fped−FROH_ was found higher in cows with CGE > 10 compared to those with CGE ≤ 10 (0.68 and 0.59, respectively; Figure 2B).

The F_ROH_ estimates and average number of ROH per cow based upon the six ROH length classes (to differentiate old and recent inbreeding) of each of the four birth-year cohorts are presented in Table 1. In the case of ROH length classes of 1–2, 2–4, and 4–8 Mbp, all the cows, regardless of birth-year cohort, exhibited some degree of inbreeding with a mean value ranging from 0.02 in the 2–4 and 4–8 Mbp in the two oldest birth-year cohorts (2002–2005 and 2006–2010) to 0.04 in the shortest length class for all the birth classes. From the 8–16 Mbp class and above, not all the cows exhibited ROH of such lengths. Only 39% of the cows exhibited ROH in the longest length class (>32 Mbp) with most of them belonging to the latest two birth-year cohorts (2011–2015 and 2016–2020) (data not shown).

**Table 1:** Descriptive statistics of inbreeding based on runs of homozygosity (ROH) divided by six length classes per each of the four birth year cohorts.

	**2002–2005 (** ***n*** **. 248)** ^**a**^			**2006–2010 (** ***n*** **. 3,883)** ^**a**^			**2011–2015 (** ***n*** **. 30,156)** ^**a**^			**2016–2020 (** ***n*** **. 40,198)** ^**a**^		
**Length Class (Mbp)**	**Mean**	**SD**	***N*.^**b**^**	**Mean**	**SD**	***N*.^**b**^**	**Mean**	**SD**	***N*.^**b**^**	**Mean**	**SD**	***N*.^**b**^**
**Inbreeding coefficients based on ROH (F**_**ROH**_ **)**												
1> ROH ≤ 2	0.04	0.01	69.4	0.04	0.01	68.7	0.04	0.01	69.8	0.04	0.01	70.4
2> ROH ≤ 4	0.02	0.01	20.1	0.02	0.01	20.9	0.03	0.01	22.8	0.03	0.01	24.0
4> ROH ≤ 8	0.03	0.01	11.4	0.03	0.01	12.6	0.03	0.01	14.1	0.04	0.01	15.6
8> ROH ≤ 16	0.03	0.01	6.10	0.04	0.01	6.98	0.04	0.01	7.88	0.04	0.01	9.07
16> ROH ≤ 32	0.02	0.01	2.12	0.03	0.02	2.67	0.03	0.02	2.96	0.03	0.02	3.38
>32	0.02	0.01	0.37	0.02	0.01	0.47	0.02	0.01	0.52	0.02	0.01	0.60
**Total**	0.15	0.03	109.5	0.17	0.03	112.5	0.18	0.03	118.1	0.19	0.03	123.2

a *n., number of animals per each birth year cohort*;

b *N., average number of ROH per animal*.

### Effective Population Size Based on Genotype and Pedigree Data

**Figure 3:**
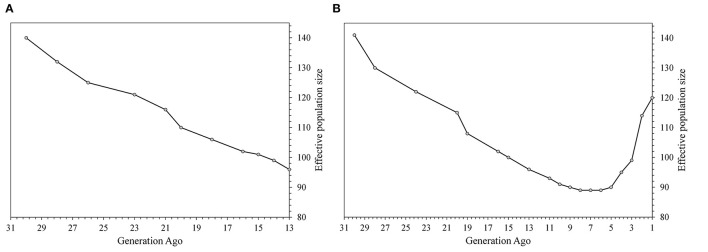
The effective population size based on SNP data calculated in the SNeP software from generation 30th using on the left **(A)** defaults settings, recombination rate according to Sved and Feldman (52) and occurrence of mutation at 2.2; on the right **(B)** a restriction on maximum distance to calculate LD, allowing estimations in the recent generations.

The two settings for the Ne estimation based on LD were comparable with an Ne reduction from generation 30 till generation 13 (Figure 3A). The Ne was equal to 140 animals at generation 30 and to 96 at generation 13 based on both settings. The second parameterization (Figure 3B) allowed the evaluation of the Ne trend at more recent generations and showed a sharp increase (almost doubled) within the last five generations, reaching to 120 in the most recent years.

The patterns of the Ne based on pedigree and SNP data were similar to each other, with differences in scale (Supplementary Figure 2). The Ne based on pedigree data was calculated from 1960 to 1964 (generation 12) to 2016–2020 (generation 1) (Supplementary Figure 2). This was equal to 87 and 55 animals in the oldest and earliest generations, respectively. A decrease in the Ne was found from generation 12 to generation 6 (1991–1995), with an Ne of 43 in generation 6. In contrast, from generation 5 (1996–2000), a general increase was observed. Likewise, for the Ne based on SNP data, a decrease was observed since generation 9 (1976–1980), followed by a plateau, with Ne equal to 89 animals from generation 8 to generation 6 (1981–1995). An increase in the Ne was observed with a value of 120 animals in the latest generation.

### The Role of GS on Genetic Diversity and GI

**Figure 4:**
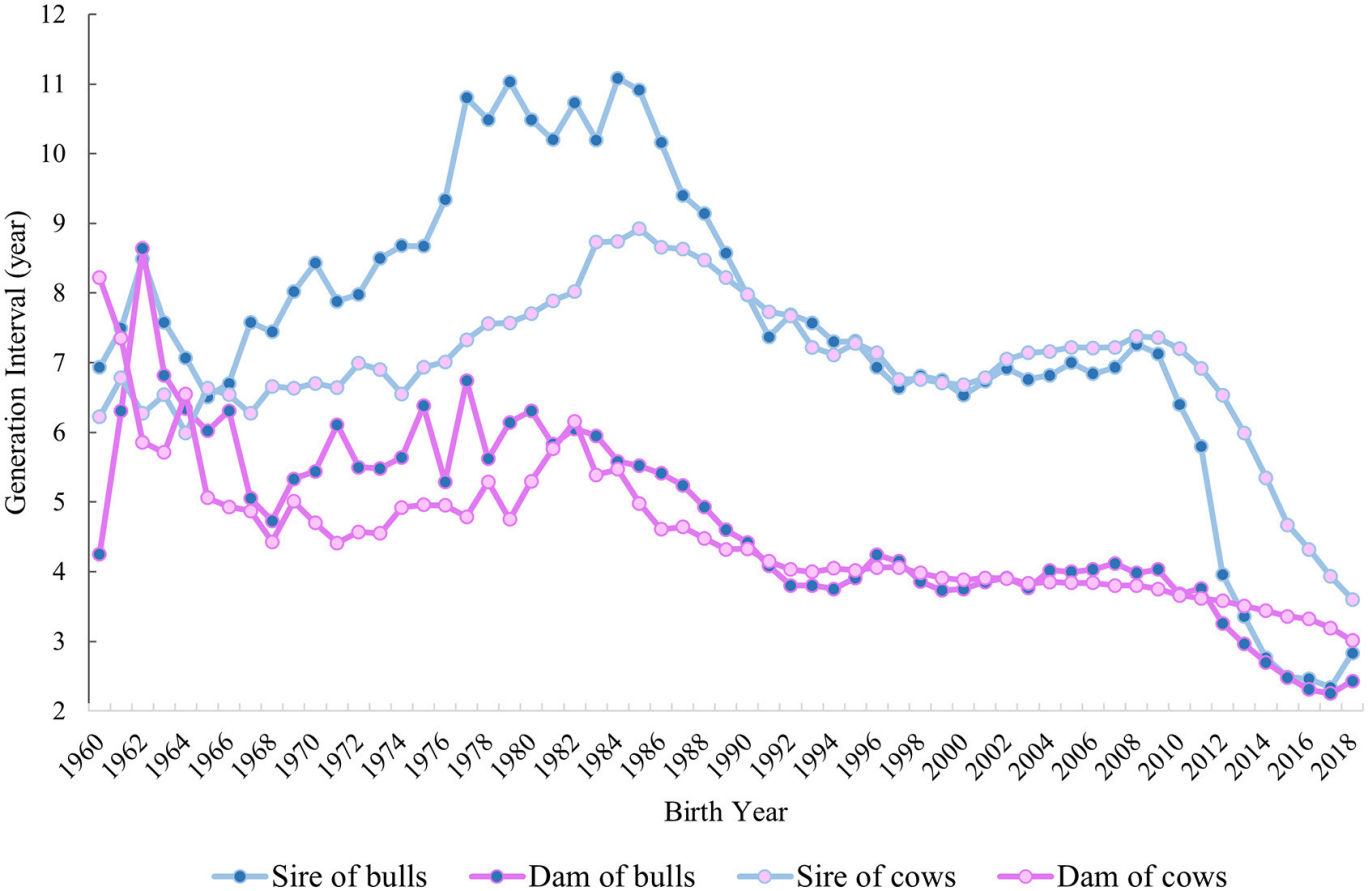
GI in years from 1960 to 2018 for the four pathways of selection: sire of bulls, sire of cows, dam of bulls, and dam of cows is shown.

GIs based on pedigree information were calculated from 1960 to 2018 for all four pathways of selection by using all animals in the pedigree file (Figure 4). For sire of bulls and cows, an increase in GI was observed till 1984, with a GI equal to 11.08 years for bulls and 8.74 years for cows. In contrast, a tendency to decrease was found from 1985 onwards for both pathways. A noticeable drop occurred in both the sire of bulls and sire of cows from 2011 to 2018, with the lowest GI in 2017 for the former (2.34 years) and in 2018 for the latter (3.6 years). The dam-of-bulls pathway decreased from 1962 (8.64 years) to 1992 (3.8 years) with some oscillations. The GI in this pathway remained approximately steady from 1993 to 2011, with a minimum of 3.68 years in 2010 and a maximum of 4.24 years in 1996, whereas it declined from 2012 to 2018 (2.43 years). In the case of the dam-of-cows pathway, a decrease was visible from 1960 (8.22 years) to 1992 (4.03 years) with few fluctuations. From 1993 onwards, the GI remained stable with an average value of 3.68 years, which varied between 3.01 and 4.0 years.

**Figure 5:**
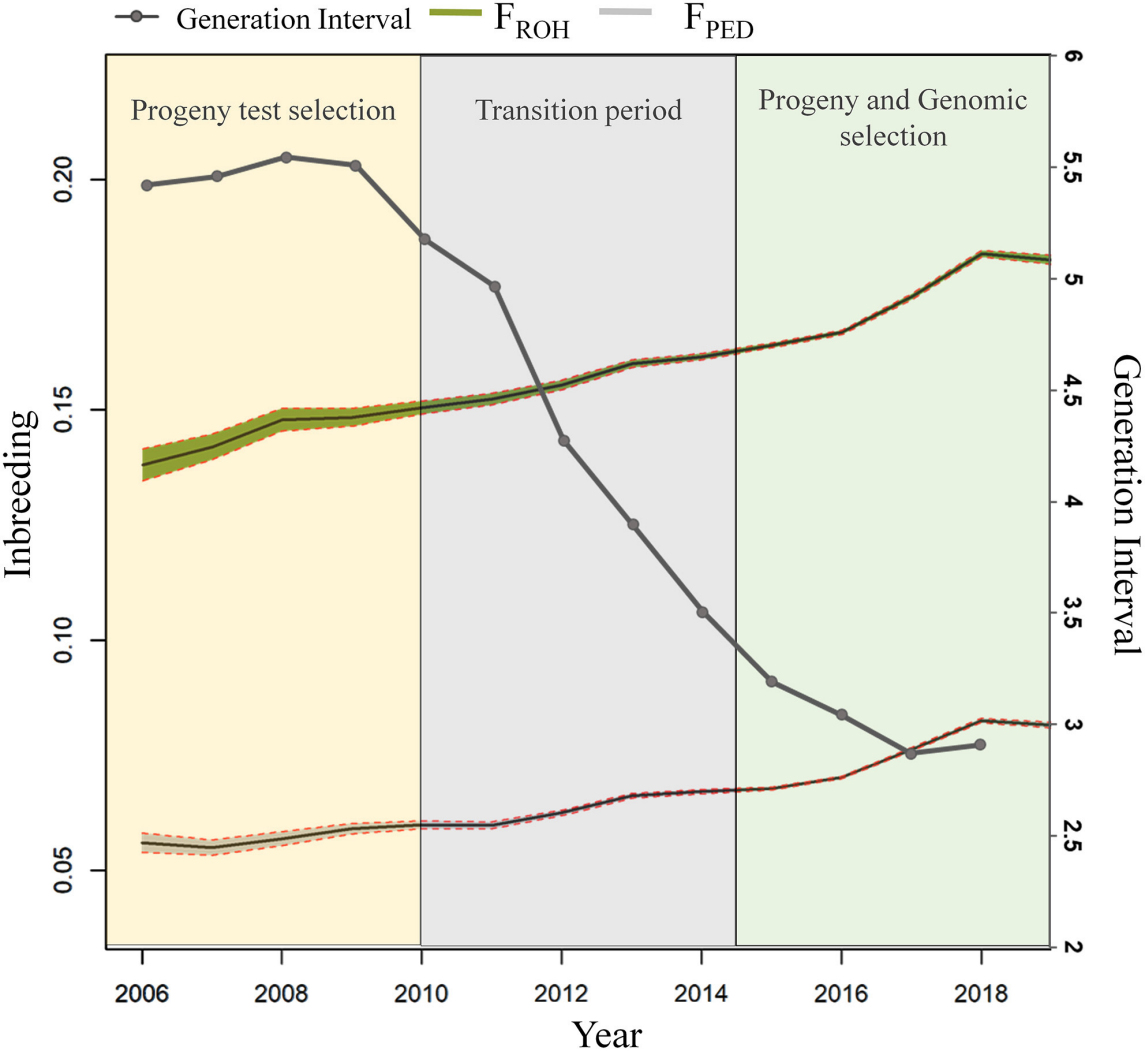
Inbreeding estimates from F_ped_, F_ROH_ and the GI between pre (called “Progeny test selection” period—from 2006 to 2010) and post the introduction of GS (called “Progeny and Genomic Selection—from 2015 to 2019) are shown.

The overall inbreeding rate per year was equal to +0.27% and +0.44% for F_ped_ and F_ROH_ throughout the studied period, which corresponds to about +1.35% and +2.2% ΔF_gen_, respectively. The mean difference in F_ped_ and F_ROH_ based on a two-sample *t*-test between the two periods (PTS and GS) was significant (*P* < 0.001) for both inbreeding estimates (Figure 5). The average F_ped_ was equal to 0.05 in the PTS and 0.07 in the GS. The average F_ROH_ was equal to 0.14 and 0.17 in the PTS and GS, respectively. The overall inbreeding rate per year in the PTS was equal to 0.14% and 0.32% based on F_ped_ and F_ROH_, whereas it increased up to 0.47% (based on F_ped_) and 0.70% (based on F_ROH_) in the GS. The RC in inbreeding comparing the two periods (PTS and GS) was equal to 2.36 and 1.19 from the F_ped_ and F_ROH_ (Table 2 and Figure 5). The overall GI (calculated as the average among the four pathways) decreased in the GS by a factor of 1.8 compared to the PTS period (Figure 5). Since this latter reduction, the ΔF_gen_ also changed between GS and PTS, being equal to +0.75% (based on F_ped_) and +1.72% (based on F_ROH_) in the PTS and 1.41 and 2.1% in the GS period, based on F_ped_ and F_ROH_, respectively.

**Table 2:** Parameters used to estimate the differences in the inbreeding trend based on pedigree and genotype data between the PTS and GS periods.

**Parameter**	**F_**ped**_**	**F_**ROH**_**
b_PTS_ (±SE)^a^	0.14% (2.0 × 10^−04^)	0.32% (4.5 × 10 ^−04^)
b_GS_ (±SE)^b^	0.47% (7.7 × 10^−05^)	0.70% (1.4 × 10 ^−04^)
δ^c^	0.0033	0.0031
*p*-value of δ-	<0.0001	<0.0001
RC^d^	2.36	1.19

a *b_PTS_ is the slope in percentage of regression per F_ped_ and F_ROH_ for cows born between 2006 and 2010 (PTS selection)*;

b *bGS - is the slope in percentage of regression per F_ped_ and F_ROH_ for cows born between 2015 and 2019 (GS)*;

c *δ is the difference between the slopes of regression of each inbreeding measurement depending on the two 5-year birth class*;

d *RC is the relative change equals to $\frac{\delta }{\beta_{1}}$ with β_1_ the slope of the second evaluated period*.

## Discussion

### Pedigree and Genotype-Based Inbreeding Coefficients

In this study, we evaluated the genetic diversity in the Italian Holstein breed by using pedigree and SNP data from cows genotyped throughout a period of 19 years. The primary goal was to estimate variation in inbreeding, effective population size, and GI and to compare those aspects prior to and after the introduction of GS in the breed. In line with the development and spread of the genotyping technology in the dairy sector (56), from 2010, the number of genotyped Italian Holstein cows per year rapidly increased, and it reached over 15,000 cows in 2016. This amount of information provided new opportunities to explore genetic diversity at the genomic level. The most traditional source of information to evaluate genetic diversity is pedigree information (48, 57). However, the accuracy of genetic diversity estimated using pedigree data highly relies on the quality and depth of the recorded data (58). In this study, the pedigree depth showed high values with an average CGE of 10.67, which agrees with previous studies on the Dutch Holstein breed (CGE between ~10 and ~14) (59, 60). Nevertheless, the CGE influenced the correlation between F_ped_ and F_ROH_. Indeed, the correlation dropped to 0.59 when considering cows with a CGE lower than 10, and it reached a value of 0.68 for animals with CGE above 10. The reduction of the correlation values for animals with lower CGE was expected, as the genealogical approach strongly depends on pedigree completeness and quality. Nevertheless, the obtained correlations are in line with the values found in previous studies, regardless of the CGE assessment. In the Spanish Holstein population, a correlation of 0.57 was shown (14), and in four different cattle breeds (Brown Swiss, Fleckvieh, Norwegian Red, and Tyrol Gray), correlations were reported to be between 0.50 and 0.72 (61). A unity correlation is not expected between F_ped_ and F_ROH_, since the former does not account for the Mendelian sampling variation, as the latter does (26, 62).

### Effective Population Size Based on Genotype and Pedigree Data

Prior to herdbook formation, the Ne was large for most cattle populations, i.e., in the order of tens of thousands (7, 63). However, the advent of closed policy in the breeding management from the nineteenth century onwards drastically reduced the gene flow among populations, causing loss of genetic diversity. The major changes in breeding programs in the last century have exacerbated this reduction. It has been shown that the current Ne in most modern and commercial dairy cattle breeds is close to 100 animals (7). Estimates of Ne found in this study based on pedigree and SNP data are in the range of those formerly published in other Holstein populations. Pedigree-based Ne estimates ranged from 39 in the US Holstein in 2000 (64) to 114 in the Canadian Holstein population between 2000 and 2007 (65). In this study, the Ne based on pedigree in 2000 was equal to 47 animals, thus slightly higher compared to the US Holstein population, yet comparable. In contrast, an over two-time lower Ne between 2000 and 2007 (Ne = 49) was found in our study compared to that reported in the Canadian Holstein population. Since 2000, there was a reduction in the inbreeding rate per generation in the Canadian Holstein (65) due to the introduction of the average relationship values in the breeding program. Since in the Italian Holstein breed this type of control was not implemented at that time, we suspect that this might be the reason behind those differences among the two Holstein populations in terms of Ne. SNP-based Ne estimates ranged from 69 in the Dutch Holstein (2) to roughly 135 in the New Zealand Holstein cattle (66). In contrast to pedigree-based inbreeding estimates, where the whole genealogical data are generally used, the SNP-based estimates are highly dependent on the genotyped animals and SNP data used for the estimation. For this reason, the comparison with other studies might be less straightforward. In addition, several methods for the Ne estimations are available based on SNP data (as an example, based on homozygous segments, marker-by-marker homozygosity, and similarity and based on LD), leading to different results. The Ne values found in the Dutch Holstein population were calculated using several SNP-based estimates (ranging between 69 and 100) (2), and most of them were lower than those found for the Italian Holstein. A possible explanation of this difference might be due to the use of different sexes in the two studies: in the Dutch Holstein (2), proven sires were genotyped, whereas in this work only females were analyzed. Thus, due to differences in the intensity of selection, we expect to have higher Ne based on female data, when compared to proven sires. New Zealand and Australian Holstein cattle (66) showed analogous estimates to the Italian Holstein in the latest generations in our study. The present study and the one by de Roos et al. (66) were based on LD-based Ne estimates, making the comparison more forthright. Nevertheless, a similar Ne across countries is expected for highly specialized worldwide breeds, due to the exchange of genetic material and similar applied breeding strategies. The Holstein fully represents this scenario as it has been bred in over 150 countries, and it currently dominates commercial dairy production worldwide (1). Although the overall breeding goal is not always uniform among countries, high selection intensities and a small number of sires are commonly used worldwide (67). In the Italian dairy industry, over 80% of bovine milk is produced by this breed, which has been used over time for a multitude of purposes, from drinkable milk to highly specialized consortia for PDO cheese production (40). This latter aspect might have caused a peculiar genomic architecture in the Italian Holstein which should be investigated further in comparison with other international Holstein populations. Nevertheless, in the Italian Holstein population, roughly 49% of the sires is local (Italian nationality), while the remaining ones are from other countries (i.e., 23% from the United States and 7% from Canada), highlighting that what we found in the Italian Holstein might resemble the status quo of other countries as well. Unfortunately, this implies that, despite the census of millions of animals, the Holstein breed is exposed to the same genetic drift and accumulation of inbreeding as a population of roughly 100 individuals. The use of a small number of sires propagated worldwide through AI and other advanced reproductive technologies, as well as mating of close relatives, has boosted this issue (65). Analysis on genetic distance and variability of different Holstein populations worldwide may provide additional information on their current relatedness, which can further serve to quantify the genetic variability in this breed at a global scale.

For the latest generation considered in this work, the Ne was equal to 55 and to 120 animals from pedigree and SNP-based estimates, respectively. The Food and Agriculture Organization of the United Nations (FAO) set the Ne critical value at 50 animals, from which the population is expected to lose fitness and variability in the long term (68). Since the Italian Holstein population seems to be very close to the critical value from pedigree analysis, appropriate strategies to reduce the loss of genetic diversity are needed to preserve genetic diversity and sustain fitness of the breed in the long term. The reduction in the Ne from 1960 to 1995 found in this study agrees with previous studies in the Canadian, Dutch, French, and US Holstein populations (65, 69). Interestingly, a similar Ne increase was observed from 2000 in the Canadian Holstein (65). This increase was even more evident in our study when looking at SNP Ne estimates, which showed an increase of about 20 animals in the latest three generations. However, the latter result seems counterintuitive if evaluated with the general increase of inbreeding over time found in the Italian Holstein. A potential reason might be the more diverse genetic pool in terms of selected candidates thanks to GS (70), leading to a reduction in LD over greater recombinant distances. With GS, we can quantify Mendelian sampling. This serves to differentiate, e.g., between full sibs, hence potentially reducing the co-selection of sibs. Thus, we expect a positive effect on Ne, which was indeed shown in this study based on both pedigree and SNP estimates. Nevertheless, we suspect that the more visible increase in Ne based on SNP compared to pedigree is due to the distinct methodologies employed based on the source of data available. In the case of Ne based on pedigree, the estimate depends on the quality and completeness of the genealogical data, whereas, in the case of SNP-based Ne, the estimate relies on the relationship between the LD variance and effective population size. Therefore, we believe that the latter estimate might be more sensible for the detection of Ne variation compared to pedigree data.

### Impact of Genomics and Future Breeding Strategies

The average GI in the Italian Holstein has decreased since 1985, with two sharp declines from 1985 to 1990 and from 2009 onwards. The initial reduction in GI might be attributed to the implementation and use of the BLUP evaluation (71). Nevertheless, from 1992 to GS, an almost constant GI was found which might be due to the general tendency of using proven bull sires. The remarkable reduction in the GI since 2009 might be attributed to GS, as predicted in a previous study (72). The severe drop in the GI found in this study is in line with reported literature. After 2009, in the Canadian Holstein, a drop of 38% in the GI was registered, as well as in the US Holstein (37%) and in the Dutch Holstein (~35%) (2, 4, 12). The ΔF_gen_ also fluctuated throughout the studied period, and it increased from 0.14 to 0.47% (pedigree based) and from 0.32 to 0.70% based on ROH comparing the PTS–GS periods. Similar rates of inbreeding were found in a previous study where over 4,000 Holstein bulls were analyzed, highlighting the effect of GS both on the annual genetic gain increase and on the inbreeding rate (13). The observed ΔF in the Italian Holstein is higher than the 1% per generation suggested by FAO guidelines as the critical value for the maintenance of genetic variability in the long term (73).

Currently, a web interface is available for breeders to evaluate and manage inbreeding within their herds, where advice on mating strategies is provided by specialists from the breeding association (74). However, the routine use of this tool by breeders is not fully known. Thus, further activities to raise awareness on inbreeding control among breeders are advised to prevent further loss of genetic variability. This latter aspect is extremely important if evaluated together with the current and coming expansion of international demand for dairy products due to emerging economies, the need for high quality milk proteins in developing countries, and world population expansion (75). In this study, we revealed loss of genetic diversity in the Italian Holstein, which might in turn cause inbreeding depression. So far, in the dairy sector, the management of genetic diversity has been kept within individual herds where ultimately breeding decisions are made. Unfortunately, this is a drawback for the implementation of one of the most effective methods to manage genetic variability, which is the optimal contribution selection (OCS) (76). Nevertheless, thanks to the genomic era, the dairy genetic industry is slowly changing, moving toward scenarios where tighter control of the population as a whole is becoming possible (i.e., vertically integrated industries) (77). Thus, the implementation of genomic OCS might prevent any further loss of genetic variability in this breed. Finally, we suggest using inbreeding estimates based on genotype data, as we have shown that they can provide a more precise evaluation of the available variability.

## Conclusion

The presented study in the Italian Holstein cattle showed the urgent matter of controlling the loss of genetic diversity in a highly specialized breed and that this loss is not enclosed to small and local breeds only. The implementation of OCS may help to alleviate this problem and prevent any further loss of genetic variability. The preservation of genetic resources is key to ensure the long-term sustainability of this breed which represents one of the most important players in the Italian dairy chain as well as to guarantee future market demands.

## Data Availability Statement

The data analyzed in this study is subject to the following licenses/restrictions: Data supporting this paper were obtained from ANAFIBJ. The genotype data are available only upon agreement with ANAFIBJ. Requests to access these datasets should be directed to jtkaam@anafi.it.

## Ethics Statement

Ethical review and approval was not required for the animal study because records used in this study were obtained from archived data from the Italian National Association of Holstein, Brown Swiss and Jersey Breeders (ANAFIBJ), and as such, no approval was required for animal experimental purposes from the Animal Care Committee unit of the University of Parma.

## Author Contributions

ASu, CC-G, and MA conceived the idea and formulated the objectives of this study. CC-G, J-TK, and GS helped in data preparation. MA conducted the analysis and wrote the first draft of the paper. ASu and ASa supervised the project. CD and GS contributed on the data visualization. ASu, CC-G, ASa, GS, RF, and CD critically reviewed the text. All authors read and approved the final manuscript.

## Conflict of Interest

The authors declare that the research was conducted in the absence of any commercial or financial relationships that could be construed as a potential conflict of interest.

## Publisher's Note

All claims expressed in this article are solely those of the authors and do not necessarily represent those of their affiliated organizations, or those of the publisher, the editors and the reviewers. Any product that may be evaluated in this article, or claim that may be made by its manufacturer, is not guaranteed or endorsed by the publisher.

## Supplementary Material

Supplementary Figure 1 Pearson correlation (above diagonal), scatterplot (below diagonal) and density (diagonal) of inbreeding coefficients measured by ROH (F_ROH_) and pedigree data (F_ped_) in the Italian Holstein dairy cows.

Supplementary Figure 2 Effective population size based on pedigree and SNP data (using the second method applied in the study for the Ne calculation) from 1960 till 2020.
