# Supplementary figures and images for: Genetic Diversity in the Italian Holstein Dairy Cattle Based on Pedigree and SNP Data Prior and After Genomic Selection
Source: Front Vet Sci. 2022 Jan 13;8:773985. doi: 10.3389/fvets.2021.773985 (PMC8792952; doi:10.3389/fvets.2021.773985)

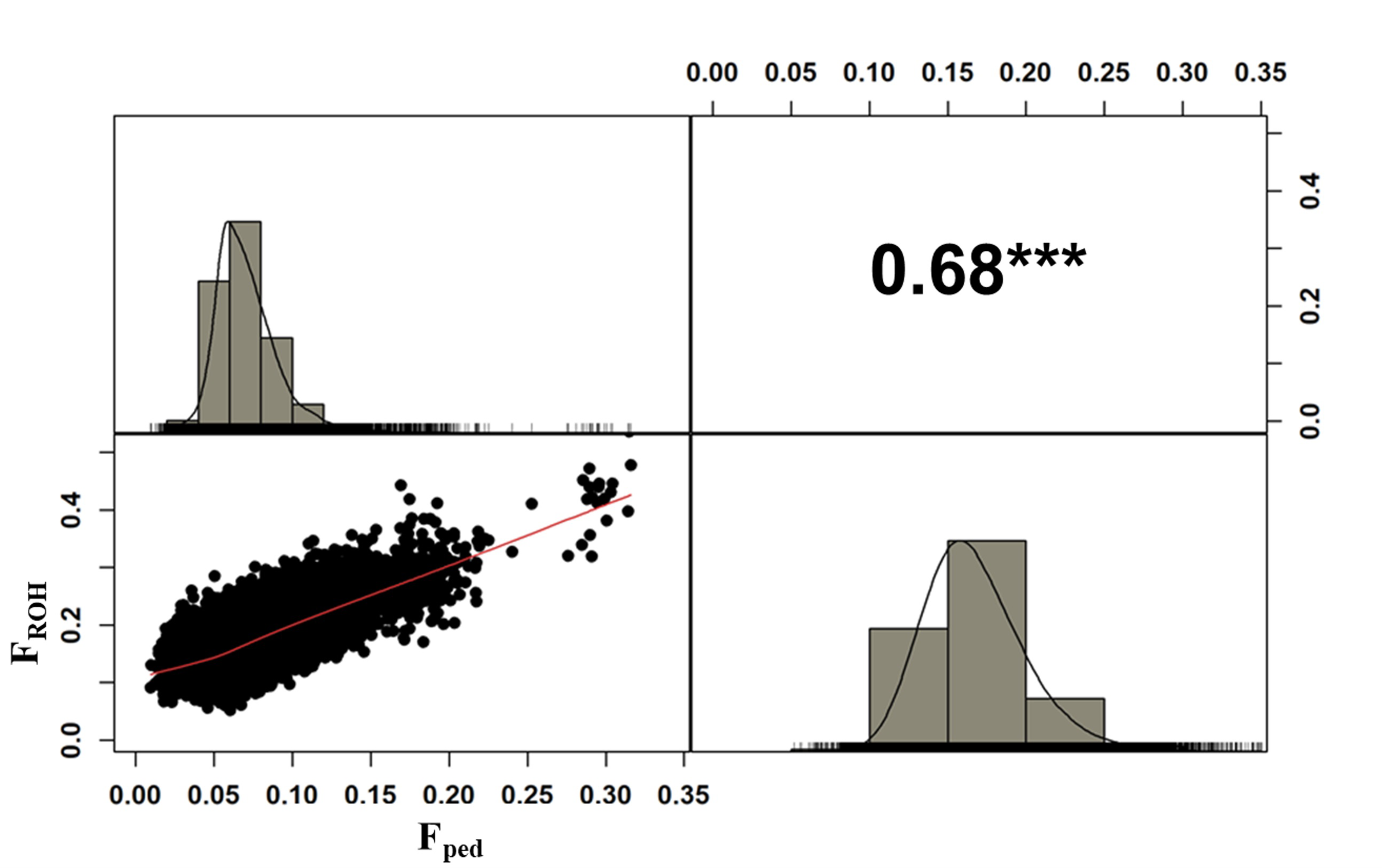

Supplement: Supplementary Figure 1 — Pearson correlation (above diagonal), scatterplot (below diagonal) and density (diagonal) of inbreeding coefficients measured by ROH (FROH) and pedigree data (Fped) in the Italian Holstein dairy cows. [file Image_1.TIF]

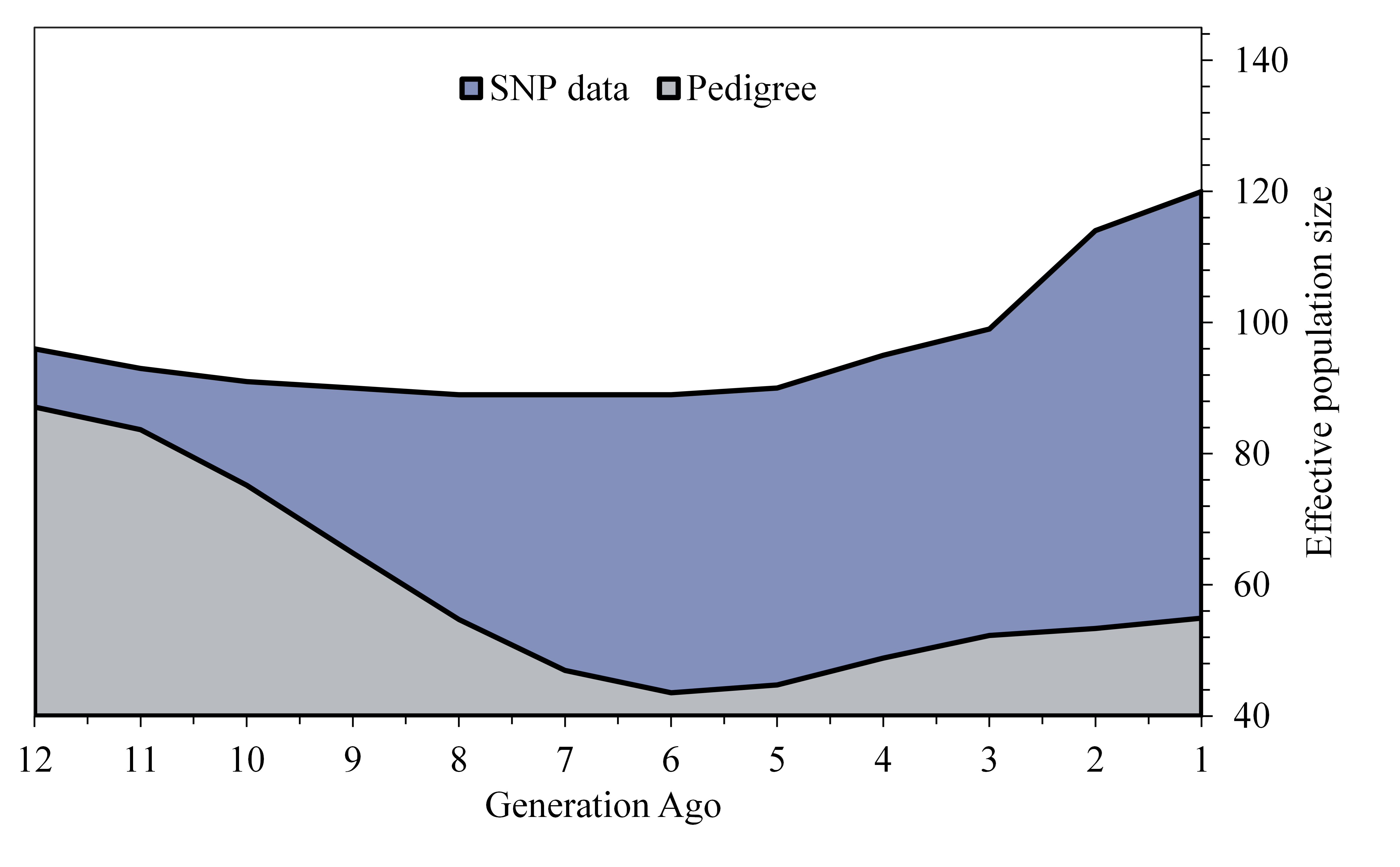

Supplement: Supplementary Figure 2 — Effective population size based on pedigree and SNP data (using the second method applied in the study for the Ne calculation) from 1960 till 2020. [file Image_2.TIF]
